# Association of temporalis muscle thickness and frailty in an Australian memory clinic cohort

**DOI:** 10.1007/s40520-025-03173-7

**Published:** 2025-08-31

**Authors:** Andrew L. H. Huynh, Sophia Avramoudas, James Andrews, Nan Jordan, Paul Yates

**Affiliations:** 1 Department of Geriatric Medicine, Austin Health, Heidelberg, VIC Australia; 2 Department of Medicine, Austin Health, University of Melbourne, Heidelberg, VIC Australia; 3 Division of Clinical Immunology and Rheumatology, Department of Medicine, University of Alabama at Birmingham, Alabama, United States of America

**Keywords:** Sarcopenia, Frailty, Temporalis muscle, Dementia, Magnetic resonance imaging

## Abstract

**Background:**

Sarcopenia and frailty are associated with cognitive impairment, and both are associated with adverse clinical outcomes. Current assessments of sarcopenia are not routinely performed in memory clinics. Temporalis muscle thickness (TMT), which can be measured on routine memory clinic brain magnetic resonance imaging (MRI), has been proposed as a surrogate biomarker of sarcopenia. However, the association of TMT and frailty has not been previously elucidated.

**Aims:**

To explore the relationship between TMT, measured on coronal T1-weighted brain MRI and frailty, as assessed using a Frailty Index (FI), in a memory clinic cohort.

**Methods:**

Retrospective cohort study of 140 patients who attended a memory clinic in a tertiary referral hospital in Melbourne, Australia in 2014. TMT and FI of patients with an adequate coronal T1-weighted brain MRI for assessment were collected. Comparisons of TMT between frail (FI ≥ 0.25) and non-frail patients were explored.

**Results:**

140 patients, median age 75.3 years old (interquartile range [IQR] 67.1–83.2 years old), 55% female. The median TMT was 5.5 mm (IQR 4.4–6.8 mm) and 34% were frail. People who were frail were more likely to be older (*p* < 0.001), have a lower MMSE (*p* = 0.003), and reduced TMT thickness (*p* = 0.011) compared to people who were not frail.

**Discussion and conclusion:**

Reduced TMT, measured in coronal T1-weighted brain MRI is associated with frailty in this cohort. Future studies incorporating additional measures of sarcopenia (e.g. DXA, dynamometry) to validate the use of TMT in coronal T1-weighted brain MRI are warranted.

## Introduction

Sarcopenia is a progressive, generalized and accelerated loss of skeletal muscle mass, quality, quantity and function, which is generally associated with age-related processes, but also occurring in mid-life in association with certain comorbid conditions [1]. Its significance rests in its association with increased incidence of adverse health outcomes, including falls, reduced ability to conduct activities of daily living, functional decline, cardiac and respiratory disease, cognitive impairment, placement in long-term care, frailty, and mortality [1]. The prevalence of sarcopenia is estimated to be 10–16% for older people globally [2]and in people with cognitive impairment, in a meta-analysis the pooled prevalence of sarcopenia was 30%, and probable sarcopenia prevalence was 41% [3].

Since sarcopenia is associated with an increased risk of a diverse range of adverse health events, it presents a potentially useful clinical parameter with which to risk stratify and prognosticate, as well as being a potentially modifiable target for intervention. However, the clinical evaluation of sarcopenia remains challenging. There is growing evidence in support of temporalis muscle thickness (TMT) as a surrogate marker of sarcopenia in Parkinson’s disease [4] glioblastoma [5] stroke [6] head and neck squamous cell carcinoma [7] and brain metastases [8]. TMT assesses muscle quality and quantity, a component of sarcopenia, and can be evaluated using brain imaging that is routinely performed in a memory clinic.

In addition to the presence of sarcopenia in individuals diagnosed with cognitive impairment, frailty is also common, with the prevalence of frailty in older adults with dementia ranging from 24–99% [9]. Frailty is a geriatric syndrome characterized by a loss of homeostatic reserve and decline across both function and body systems [10]. Similar to sarcopenia, frailty is associated with increased adverse health outcomes including falls, fractures, hospital admissions, disability, long-term care admission and mortality [10]. Though there are distinct differences with sarcopenia, the physical component of frailty specifically overlaps with definitions of sarcopenia; and certain interventions, including optimization of protein intake and prescribing of physical exercise, are common to both [1]. However, the relationship between TMT and frailty has not been previously elucidated.

To better understand the overlap between sarcopenia and frailty, and how one may contribute to the other, we aimed to explore the relationship between TMT using coronal T1-weighted brain magnetic resonance imaging (MRI) and frailty, as assessed using a frailty index (FI), in a memory clinic cohort. It was hypothesized that increased TMT would negatively correlate with frailty.

## Methods

### Study design

Cross-sectional study of a retrospective cohort of patients who attended the memory clinic in 2014 at Austin Health, a tertiary referral hospital in Melbourne, Australia. The Cognitive, Dementia and Memory Service (“Memory Clinic”) at Austin Health is a diagnostic clinic, where referrals for patients are typically from their primary care physician, but they can also be self-referred, for diagnosis of cognitive concerns. Patients with a previously established diagnosis of dementia are referred to a different clinic for ongoing management, and so were not included in this study. Patients in the Memory Clinic undergo a clinical, cognitive, physical, and neurological assessment, brain imaging (computerized tomography [CT]/MRI), single-photon emission CT (SPECT) and positron emission tomography (PET) scans. A consensus cognitive diagnosis is made in a multidisciplinary case conference compromised of geriatricians, a neurologist, a nuclear medicine physician, geriatric medicine trainees and neuropsychologists. The inclusion criteria for the study were that the patient underwent a brain MRI as part of their workup, that the coronal MRI of the brain was of an adequate image quality and definition for morphometric analysis, and sufficient data was available to derive a FI.

### Data collection

Data collected included age in years, gender, mini-mental state examination (MMSE) and Rowland universal dementia assessment scale (RUDAS) score at memory clinic, cognitive diagnosis, FI and TMT. The cognitive diagnosis was categorized as either normal/subjective cognitive impairment, mild cognitive impairment (MCI), dementia or other impairment (e.g. acquired brain injury/psychiatric/obstructive sleep apnea). The collection of FI and TMT are described below.

#### Frailty index (FI)

The FI measures frailty as a ratio between health deficits present in the individual, and the total number of health deficits measured, which is constructed following the guidelines by Searle et al. [11]. The 54 variables used in the FI collected in this study are described in Table 1, and include medical comorbidities, medication use, social support, functional status, recent falls, disability and physical parameters such as blood pressure and weight loss. There were no intermediate values in the variables used to construct the FI (0 = health deficit not present, 1 = health deficit present). The FI was constructed based on retrospective chart review from the hospital’s electronic medical records of referral and outpatient letters, memory clinic medical reviews, allied health assessments and multidisciplinary case conference reviews within 12 months prior to radiological assessment. Frailty was defined as a FI ≥ 0.25, as has been previously published [11] where a FI score of 0 denoted no frailty and 1 would denote maximum frailty.

**Table 1:** Frailty index (54 health deficits)

Help dressing	Eyesight issues	Skin problems
Help bathing	Hearing issues	Other musculoskeletal conditions
Help grooming	Dental problems	Other medical issues
Help toileting	Eye problem	Lives alone
Help eating	Ear problem	Weight loss > 4.5 kg in the last year
Help using phone or remote control	Head and neck issues	Impaired mobility/mobility aids
Help with housework	Chest problems	Systolic BP < 90 or > 140
Help with finances	Chronic lung disease	Diastolic BP < 60 or > 90
Help with meal preparation	Respiratory issues	Sleeping issues
Help taking medications	Gastrointestinal problem	Thyroid issues
Help with shopping	Constipation	Hypertension*
Help to get to places out of walking distances	Abdominal issue	Hyperlipidaemia*
Medications > 5	Malignancy	Myocardial Infarction*
> 1 Falls in the last year	Osteoporosis	Congestive Heart Failure*
Urinary incontinence	Arthritis	Arrhythmia*
Bowel incontinence	Spinal issues	Vascular issues*
Rectal problems	Trouble with feet or ankles	Chronic renal disease*
Genito-urinary problems	Trouble with nerves	Diabetes Mellitus*

*Health deficits classified as vascular risk factors

Head and neck issues: any medical diagnosis related to the head or neck that did not involve the eye, ear, teeth or skin

Chest problems: symptoms involving the chest that were not respiratory symptoms/chronic lung disease/myocardial infarction/congestive heart failure/arrhythmia and did not fit under any other health deficit

Trouble with feet or ankles: symptoms relating to feet or ankles that were not arthritis/osteoporosis and did not fit under any other health deficit

Trouble with nerves: neurological symptoms that did not fit under any other health deficit

Other medical issues: any medical diagnosis that did not fit under any other health deficit

#### Radiological assessment

All MRIs of patients were scanned on a single 1.5T Siemens Avanto scanner (Siemens, Erlangen, Germany), producing slices of 2.5 mm thickness (flip angle, 15°; echo time, 40 ms; and repetition time, 27 ms) at the Department of Radiology. Imaging acquired included the axial susceptibility-weighted image (SWI), 3D T1/MPRAGE, coronal T1 MRI, and axial T2/FLAIR (Fluid-attenuated inversion recovery) following a standard “Memory Clinic” protocol. Axial T1 images were also initially provided, however during the study period this was removed from the routine imaging protocol and so were not available for all patients.

TMT was analyzed on coronal T1-weighted brain MRI and axial T1-weighted brain MRI if available. The orbital roof and Sylvian fissure were utilized as anatomical landmarks. Measurements were taken perpendicular to the long axis of the muscle. TMT was measured bilaterally, and the average TMT was used for analysis. Muscle measurements were analyzed blinded to patient identifiers, clinical information and FI.

### Statistical analysis

All patient characteristics were tabulated showing frequencies and percentages for categorical variables and median/interquartile range given non-normal continuous data. The two groups of frail and non-frail patients were compared using the Mann-Whitney U test, and chi-square χ^2^ test for categorical data. Spearman correlation (ρ) was used to investigate the relationship between TMT and FI and the relationship between the average coronal and axial TMTs. A *p* value of less than 0.05 was considered statistically significant. All analyses were performed using Stata/IC v16.1 (StataCorp 2019. College Station, TX, USA: StataCorp LLC).

## Results

There were 209 patients who attended the memory clinic in 2014. Of these 140 (67%) patients had a brain MRI with adequate coronal T1-weighted MRI for TMT measurements. Characteristics of patients are shown in Table 2. The median age of the patients was 75.3 years old (interquartile range [IQR] 67.1–83.2 years old), with 55% (77 patients) women. 127 (91%) patients had an MMSE performed with the median score 26 (IQR 23–29). 14 (10%) patients had a RUDAS completed (median 23 [IQR 16–26]). 15 (11%) patients had both MMSE and RUDAS performed, while 8 (6%) patients did not have either cognitive test done (4 dementia, 4 MCI). In terms of primary diagnosis, 35 (25%) patients received a diagnosis of MCI, and 45 (32%) patients received a diagnosis of dementia. 11 (8%) patients received a formal diagnosis of another medical or psychiatric condition, such as acquired brain injury (ABI), obstructive sleep disorder (OSA), and depression, amongst others. 49 (35%) patients were classed as subjective cognitive impairment.

**Table 2:** Characteristics of memory clinic patients at Austin health in 2014 who had an MRI brain by frailty

	Total (*n = 140*)	Not Frail (FI < 0.25) (*n = 92*)	Frail (FI ≥ 0.25) (*n = 48*)	*p*-value
Age (years)	75.3	71.2	79.1	< 0.001
	(66.5–75.3)	(63.4–79.0)	(74.2–84.6)	
Female	77 (55)	49 (53)	28 (58)	0.567
Frailty index	0.18	0.14	0.32	< 0.001
	(0.11–0.27)	(0.09–0.18)	(0.27–0.35)	
MMSE (n = 127)	26	26	24	0.003
	(23–29)	(24–29)	(22–27)	
		(*n* = 85)	(*n* = 42)	
RUDAS (n = 14)	23	24	17	0.079
	(16–26)	(23–26)	(11–21)	
		(*n* = 8)	(*n* = 6)	
*Cognitive diagnosis*				0.112
Normal/subjective cognitive impairment	49 (35)	35 (38)	14 (29)	
Mild cognitive impairment	35 (25)	22 (24)	13 (27)	
Dementia	45 (32)	25 (27)	20 (42)	
Other (Acquired brain injury/ psychiatric/obstructive sleep apnoea)	11 (8)	10 (11)	1 (2)	
Mean temporalis muscle thickness (mm)	5.5	5.7	4.8	0.011
	(4.4–6.8)	(4.7–6.9)	(4.1–6.4)	

Data are n (%) and median (interquartile range, IQR) unless specified otherwise. FI = frailty index, MMSE = mini-mental state examination, RUDAS = Rowland universal dementia assessment sca

**Fig. 1:**
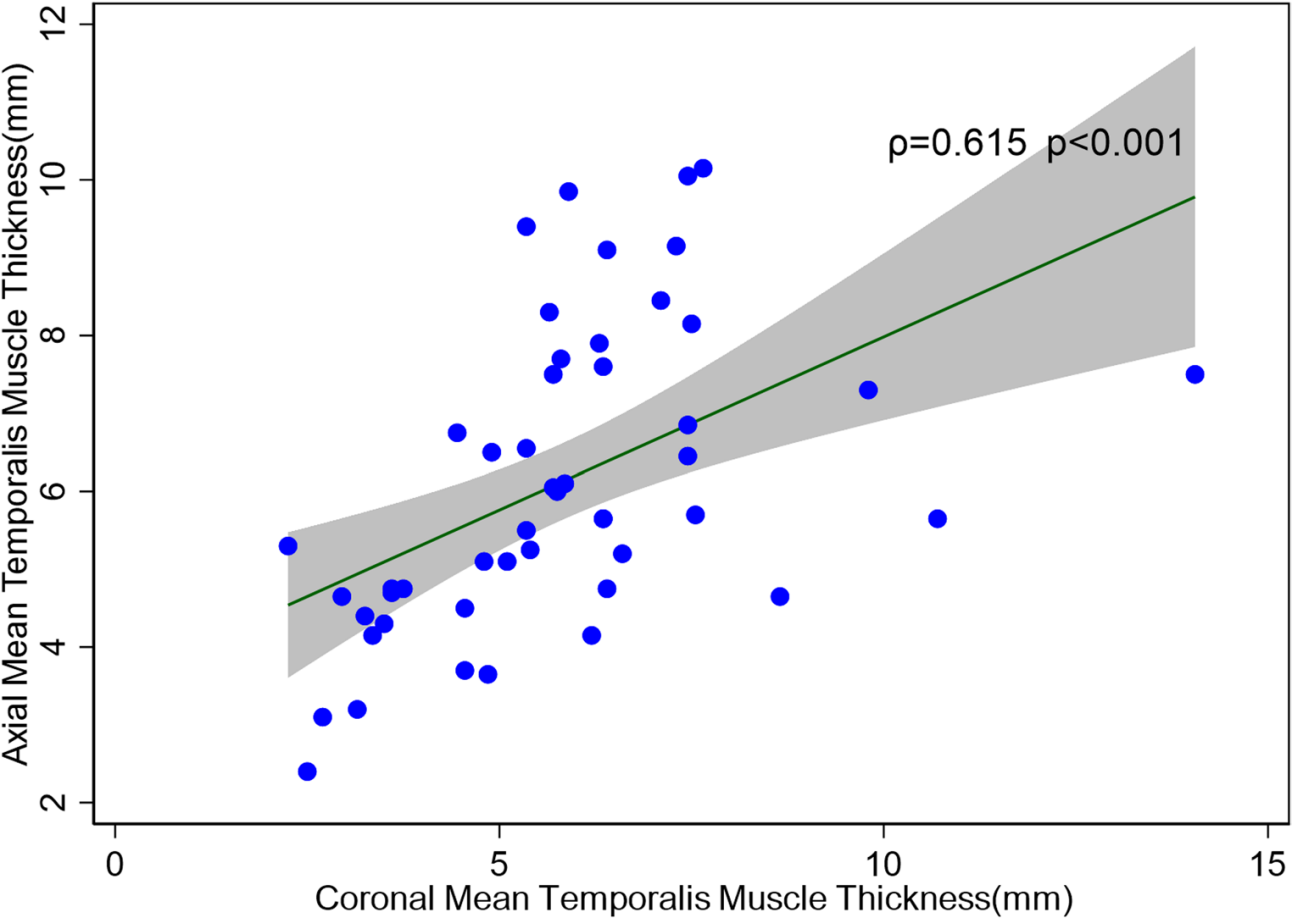
Scatterplot of coronal mean temporalis muscle thickness (mm) vs. axial mean temporalis muscle thickness (mm) ρ = Spearman correlation

While all patients had a coronal T1-weighted MRI, 49 (35%) patients also had an axial T1-weighted MRI sequence available. There was moderate positive correlation between the TMT measured in axial images compared to coronal images (ρ = 0.615, *p* = < 0.001) (Fig. 1).

**Fig. 2:**
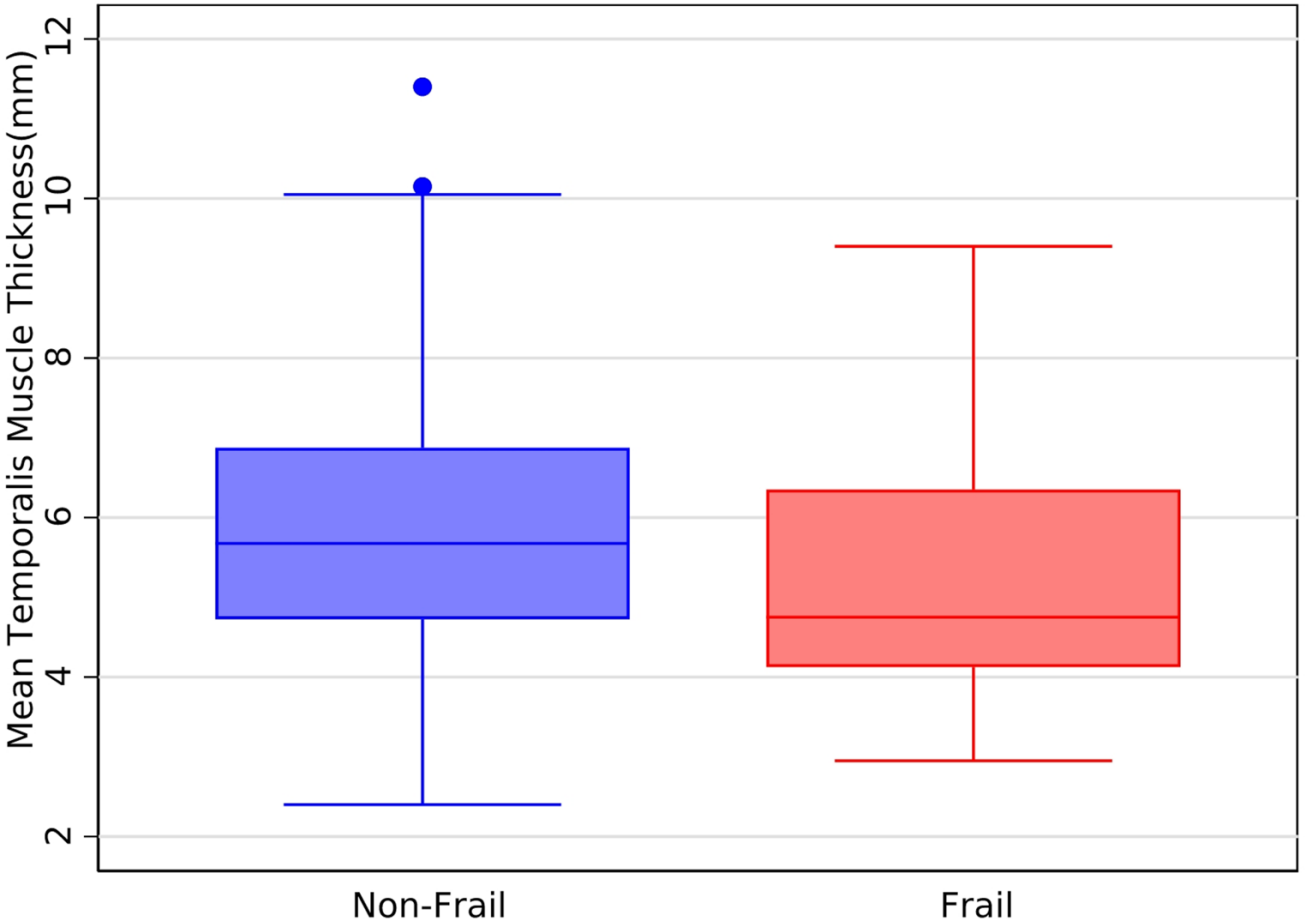
Boxplot of coronal mean temporalis muscle thickness by frailty status (*p* = 0.011)

**Fig. 3:**
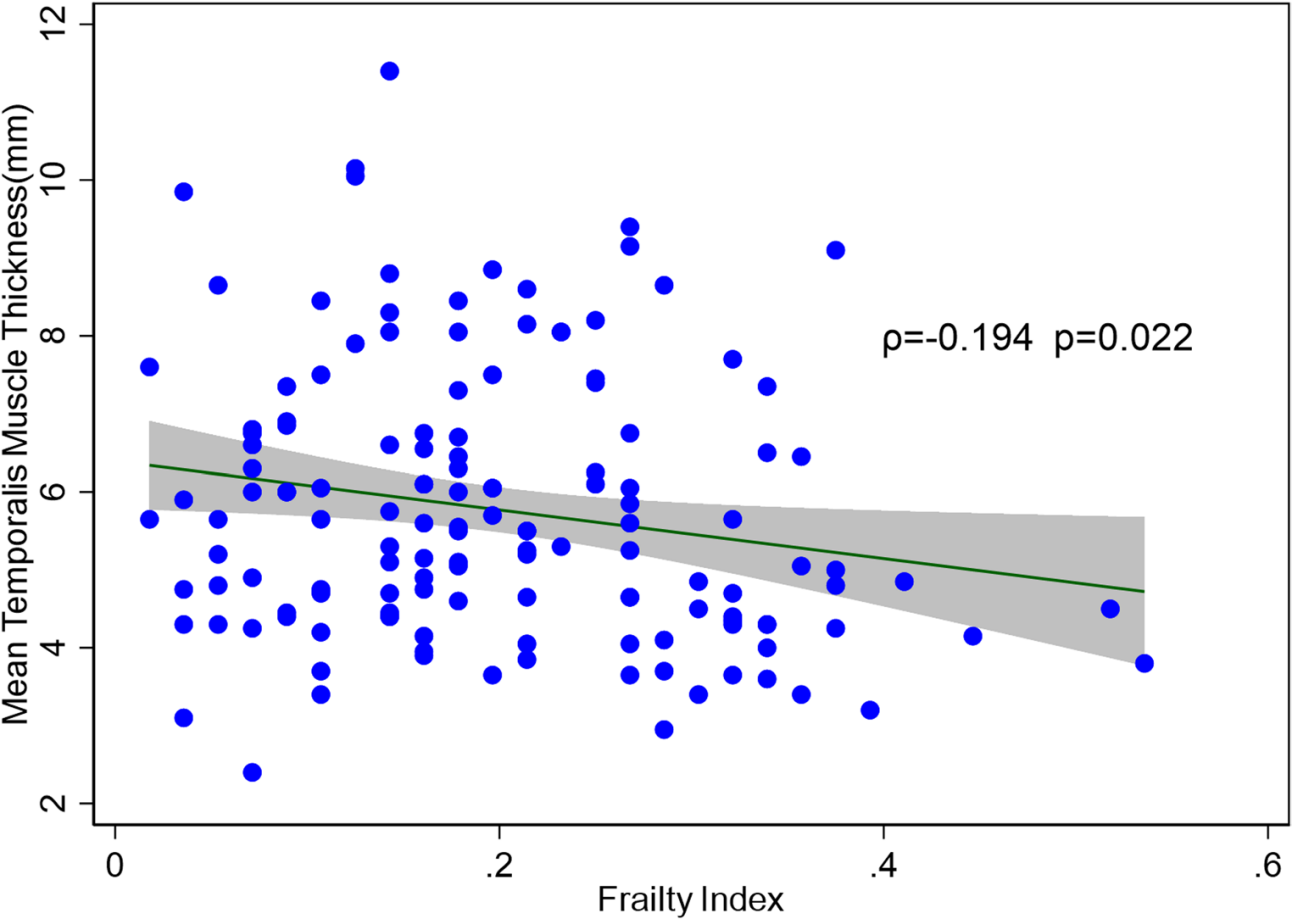
Scatterplot of coronal mean temporalis muscle thickness (mm) by frailty index ρ = Spearman correlation

The median coronal mean TMT was 5.5 mm (IQR 4.4–6.8 mm.). The median FI was 0.18 (IQR 0.11–0.27). Based on the pre-established cut-off of FI ≥ 0.25, 48 (34%) patients were frail. A comparison of coronal TMT between frail and not frail people is shown in the box plot in Fig. 2. People who were frail were more likely to be older, have a lower MMSE, and reduced TMT thickness compared to people who were not frail (Median age – frail 79.1 years old [IQR 74.2–84.6 years old] vs. not frail 71.2 years old [IQR 63.4–79.0 years old] *p* < 0.001; median MMSE – frail 24 [IQR 22–27] vs. not frail 26 [24–29] *p* = 0.003; TMT – frail 4.8 mm [IQR 4.1–6.4 mm] vs. not frail 5.7 mm [IQR 4.7–6.9 mm] *p* = 0.011 ). There was weak negative correlation between TMT and frailty as assessed using FI as a continuous variable (ρ = -0.194, *p* = 0.022) (Fig. 3).

## Discussion

In this study of 140 memory clinic patients, people who were frail were more likely to be older, have a lower MMSE, and reduced TMT compared to people who were not frail. TMT had weak correlation with frailty when FI was used as a continuous variable.

To our knowledge, this is the first study to examine the use of TMT measurement on coronal MRI in patients with memory complaints. While most previous studies have used axial MRIs to measure TMT, our study only had access to axial T1-weighted MRIs for 35% of patients. To address this limitation, we used coronal MRIs, which are commonly used in a memory clinic context to assess for hippocampal atrophy e.g. in Alzheimer’s disease. There was (significant) moderate positive correlation between axial and coronal T1-weighted brain MRI to measure TMT. Since coronal T1-weighted MRI is a routine investigation in this cohort, this provides a more readily-available opportunity for measurement of TMT, potentially representing a practical method to assess muscle quantity and quality without requiring additional investigations or sequences. However, further correlation of TMT from coronal T1-weighted brain MRI with gold-standard measurements of muscle quantity and quality such as skeletal appendicular muscle mass performed by dual-energy X-ray absorptiometry (DXA) or bioelectrical impedance analysis (BIA) are required in future studies. Assessment of correlation between TMT from axial-T1-weighted brain MRI and gold-standard muscle quantity and quality measurements have been previously performed. A study by Cho et al. [12] showed low positive correlation between axial T1-weighted TMT and skeletal appendicular muscle mass (measured by DXA) in a small study of 68 patients with Alzheimer’s disease in Korea (*r* = 0.379, *p* = 0.001). Leitner et al. [8] found strong correlation of axial T1-weighted TMT and the cross-sectional area of abdominal muscles at the L3 vertebrae on CT images in 154 patients (93 lung cancer, 61 melanoma) with brain metastases (*r* = 0.733, *p* < 0.001). Another area of interest in the future might be to compare TMT to clinical parameters of muscle function such as the timed up and go test and grip-strength dynamometry, as these may more accurately act as a comparator for sarcopenia specifically, as well as provide useful information as to the association between TMT and functional outcomes. Further studies are also required to corroborate the correlation of TMT measurement between T1-weighted axial and coronal images and enhance the generalizability of our study.

TMT (using axial T1-weighted MRI) was associated with mortality in people with Alzheimer’s disease and Lewy body dementia, and cognitive performance and functional decline in Lewy body dementia [13]. However, it is unknown if these findings also hold if TMT is measured in the coronal plane. Post-mortem studies suggest that frailty influences the clinical expression of dementia [14]. Future studies could longitudinally evaluate the relationship between frailty, TMT and other long-term adverse health outcomes in clinic cohorts, such as the rate and degree of functional decline and the time to falls, fractures, hospitalization, permanent residential aged care, and death.

Our study has several limitations. Although the results suggest an association between TMT and frailty using a defined cut-off of FI ≥ 0.25, the association was attenuated when FI was used as a continuous variable. This limits the viability of TMT as a tool for clinician use in this cohort, and may be attributed to several factors. While the FI is a convenient measure of frailty in a research context, and can be constructed from patient data retrospectively, it can be cumbersome to use in clinical practice. Given the time difference between FI collection and imaging exam, there could be information bias; it is possible that individuals may have accumulated additional health deficits between clinical review and MRI scan. In addition, the frail cohort was older than the non-frail cohort in our study, raising concerns of bias in our findings. Given the relatively small sample size, we aimed to look at the strength and direction between TMT and FI only, as the study was not powered to conduct a multivariable regression analysis. A post-hoc sample size calculation demonstrated that for a small effect size (Cohen d = 0.20), with three variables in model (TMT, age, sex), α = 0.05, β = 0.80, a sample size of approximately 277 would be required. Future studies with larger sample sizes should confirm the use of coronal T1-weighted TMT using regression analysis, ideally adjusting for age, sex, and other confounders. It is also possible that the most frail people did not receive MRI due to perceived test burden or contraindications; future research using CT imaging could extend this work to a broader population. Our study is also limited in its retrospective nature, and its single center focus, raising concerns of selection bias. Future studies could aim to have a larger cohort of patients from multiple memory clinics using coronal T1-weighted MRIs. Given that the temporalis muscle is involved in mastication, the presence of ill-fitting dentures of extensive dental procedures could have an impact on mastication and thus TMT. While our study collected data on dental problems as part of the frailty index, we did not collect information such as the presence of dentures or if the individual had extensive dental procedures. Our study only assessed patients at the time of cognitive diagnosis, and this selection bias could suggest that our findings are not generalizable to individuals living with a diagnosis of dementia for varying lengths of time. Future studies that assessed longitudinal follow-up of patients post memory clinic review with brain magnetic resonance imaging and frailty assessment are required to assess the relationship between sarcopenia and frailty.

The strengths of our study are that we used real world data of patients who presented to a single hospital memory clinic in Australia, with detailed clinical characterization and robust consensus diagnostic confirmation. All patients were scanned on the same MRI camera, and received an imaging protocol typical of a diagnostic cognitive workup, and ascertainment of the chosen muscle measure did not require additional equipment or increase the burden for patients with cognitive impairment and their carers.

## Conclusion

The findings were that TMT can be reliably and conveniently measured from coronal T1-weighted MRIs obtained from a memory clinic cohort, and that there is an association between TMT and frailty as assessed using FI. Increased TMT is associated with decreased frailty in this cohort. Future studies might seek to explore the relationship between TMT and long-term clinical and functional outcomes, corroborate the moderate correlation in TMT measurement between T1- weighted axial and coronal sequences, and explore the relationship between TMT and gold-standard measures of sarcopenia.

## Data Availability

The data can be obtained from the corresponding author upon reasonable request.
